# Global patterns linking total meat supply to dementia incidence: A population-based ecological study

**DOI:** 10.3934/Neuroscience.2025012

**Published:** 2025-06-05

**Authors:** Wenpeng You

**Affiliations:** 1 Adelaide Medical School, the University of Adelaide, Adelaide, Australia; 2 Adelaide Nursing School, the University of Adelaide, Adelaide, Australia; 3 School of nursing and Midwifery, Western Sydney University, Sydney, Australia

**Keywords:** meat supply, dementia, global health, aging, diet

## Abstract

Dementia cases are projected to triple globally by 2050, largely driven by an aging population. While aging remains the primary risk factor, emerging evidence suggests that diet, including total meat supply, may influence dementia risk. This study investigates the relationship between total meat supply (red and white meat) and dementia incidence using data from 204 countries. Bivariate correlations revealed a significant positive association between total meat supply and dementia incidence globally (r = 0.59, p < 0.001), with a stronger effect observed in low- and middle-income countries (z = 3.92, p < 0.001). Partial correlation analyses and multiple regression models, controlling for aging, economic status, genetic predisposition, and urbanization, confirmed that meat supply remained a significant predictor of dementia (Beta = 0.20, p < 0.001). Aging showed the strongest influence (Beta = 0.79, p < 0.001), underscoring its dominant role. Regional analyses suggested socio-economic disparities, dietary habits, and limited access to diverse nutrition as factors amplifying the association in developing regions. These findings identify total meat supply as a modifiable dietary factor contributing to dementia risk, particularly in resource-constrained settings. Implementing tailored dietary interventions may help reduce dementia incidence globally, especially in vulnerable populations.

## 1. Introduction

Dementia is a pressing global health issue affecting millions, with its prevalence expected to rise substantially due to an aging population. The World Health Organization (WHO) predicts that by 2050, the number of people living with dementia will triple [1]. This neurodegenerative condition is marked by a progressive decline in cognitive abilities, including memory, reasoning, and behavior [2]. Although aging remains the most critical risk factor, there is an increasing focus on modifiable lifestyle influences, particularly dietary factors, that may contribute to dementia onset and progression [3]. Among these, meat supply has emerged as a potential contributor to dementia risk, though its role remains under-investigated [4].

Dietary habits significantly impact health outcomes, with ample evidence linking certain diets to reduced risks of chronic diseases. Research on diet and dementia has predominantly centered on dietary patterns, such as the Mediterranean diet, which is predominantly plant-based and rich in whole grains, vegetables, fruits, and healthy fats [5]. These diets have been associated with lower dementia risk due to their anti-inflammatory and cardioprotective properties [6]. For instance, the Mediterranean diet supports vascular health, which is vital for cognitive function maintenance [7]. However, the potential influence of meat consumption, especially total meat supply, on dementia risk has received comparatively little attention.

In recent decades, global meat supply has surged, doubling or even tripling, particularly within developing regions [8]. This increase has coincided with a rise in dementia prevalence in several low- and middle-income nations, such as China, Peru, and Cuba, as suggested by ecological and cross-sectional studies [9],[10]. In Sweden, research involving cognitively healthy adults found that lower meat supply correlated with improved cognitive function and greater brain volume over a five-year period [11]. Recent studies have further strengthened the evidence linking long-term red meat consumption to dementia risk and cognitive decline [12], while systematic reviews underscore the protective role of Mediterranean and Nordic dietary patterns [13]. However, the specific effects of different meat types on dementia risk remain inadequately explored, with findings still limited and inconsistent [14],[15].

Most existing research has concentrated on individual meat types, such as beef, pork, and lamb [14]. There is a need to differentiate between processed and unprocessed meats, as these forms may have distinct health effects [16]. A systematic review on meat consumption and cognitive disorders, including dementia, found that studies often analyzed meat supply as part of broader dietary patterns, resulting in high variability and limited clarity on the effects of specific meat types or quantities [14].

The focus on red meat alone may overlook the broader dietary context. Populations generally consume both red and white meats, and studies focusing on total meat supply may provide a more comprehensive understanding of potential cognitive health risks [17]. Although white meat, such as poultry, is often considered a healthier alternative to red meat due to its lower saturated fat content, recent findings suggest it may also carry cognitive health risks. White meat contains L-carnitine, a compound metabolized by gut bacteria to produce trimethylamine-N-oxide (TMAO) [18], which is implicated in neuroinflammation, a critical factor in dementia development [19].

In addition to TMAO, both red and white meats contain other compounds that may impact cognitive health [19]. For example, heme iron, primarily found in red meat, can induce oxidative stress, while advanced glycation end products (AGEs) [20], which accumulate in cooked meats, are linked to inflammation and neurodegeneration. Although culinary classifications categorize red and white meats based on color and texture, there is substantial overlap in their biochemical compositions [21]. For instance, iron, a factor associated with dementia risk, is present in both red and white meats, though in different amounts. These factors suggest that total meat supply may be more relevant to dementia risk than red meat alone. Nevertheless, few studies have rigorously examined the role of overall meat consumption in dementia, highlighting a critical research gap [22].

This study investigates the hypothesis that high meat consumption may contribute to increased dementia incidence globally. Addressing a gap in the current literature, this research evaluates whether total meat supply serves as a predictor of dementia risk. Building on prior studies that link high meat consumption to various chronic health conditions, this study explores whether similar patterns appear in relation to dementia. By leveraging global data, the analysis examines the relationship between meat supply and dementia while controlling for factors such as economic status, age, genetic predispositions, and urbanization.

## 2. Materials and methods

### 2.1. Data collection and selection

For this population-level analysis, data were gathered from recognized international sources, including United Nations (UN) agencies and the Institute for Health Metrics and Evaluation (IHME). The dataset on dementia incidence was specifically sourced from IHME [23], while a comprehensive list of 204 regions was obtained from the World Bank to ensure consistency in variable matching. In this context, the term “country” refers to a geographic region that reports separate health, demographic, and economic data, as defined by international organizations such as the World Bank. This designation does not necessarily imply political sovereignty, and the terms “country” and “population” are used interchangeably in this study [24].

The primary independent variable, meat supply, was measured using the average total meat supply per capita over the period 2019 to 2021, to approximate medium-term dietary exposure. These data were sourced from the FAOSTAT food balance sheet (FBS) and expressed in kg per capita per year [25]. This measure reflects the average quantity of animal-based food available per individual, encompassing both red and white meats. The included types were beef, veal, buffalo, pork, mutton, lamb, goat, horse, poultry (i.e., chicken, goose, duck and turkey), rabbit, game, and offal [25]. It is important to note that FAO food supply data reflect the average quantity of food available for consumption at the national level and not the actual amount consumed. Studies estimate that approximately 70% of this supply is typically consumed, with the remaining 30% lost to waste, spoilage, or inefficiencies in storage and distribution. The initial literature review was conducted in 2022 using databases such as PubMed, Scopus, and Google Scholar. Additional searches were conducted in May 2024 and May 2025 to capture recently published studies relevant to meat consumption and dementia or Alzheimer's disease risk.

The dependent variable, dementia incidence rate (new cases per 100,000 individuals), was taken from IHME's 2021 dataset. IHME [23], an independent research institute within the University of Washington, is noted for its comprehensive work in global health statistics and its data-driven strategies addressing health challenges.

Given dementia's multifactorial aetiology, this study accounted for potential confounding variables that could influence the observed relationship between meat supply and dementia incidence. Economic status, quantified through per capita gross domestic product (GDP) adjusted for purchasing power parity (PPP) in 2018, was one such factor sourced from the World Bank database. Economic affluence correlates with longer life expectancy, higher education levels, and lifestyle-related risk factors like obesity and diabetes [26]. It also influences the capacity for early dementia detection, reflecting variations in healthcare infrastructure globally [27].

The Biological State Index (I_bs_) is used as a proxy for genetic predisposition, reflecting the accumulation of dementia-related genetic traits within a population. This index, ranging from 0 to 1.0, was obtained from a 2022 study [1]. The underlying concept is that reduced natural selection may allow the persistence of deleterious genes, thereby increasing the risk of non-communicable diseases such as dementia [28]. Populations with higher I_bs_ scores are considered to carry a greater genetic vulnerability to these conditions [29]. Although the acronym “I_bs_” does not follow the conventional word order, it has been retained to remain consistent with the original terminology used by Henneberg and colleagues in the source publication [30],[31].

Life expectancy at birth, serving as a proxy for population aging, was obtained from the World Bank database [32]. While dementia can manifest at various life stages, it primarily affects older adults; hence, 2018 life expectancy data were used to represent the aging process in this analysis. Data on urbanization, defined as the proportion of the population residing in urban areas in 2018 [26], were also sourced from the World Bank. Urban environments can shape lifestyle behaviors, influencing choices that affect dementia risk. Modernization and industrial growth have been linked to lifestyle shifts such as increased meat consumption [33],[34], greater availability of processed foods rich in salt, sugar, and fats [35], and reduced physical activity [36]. These factors collectively contribute to health outcomes relevant to dementia. However, urban settings may also facilitate earlier detection and diagnosis, potentially affecting reported incidence rates and adding complexity to dementia data interpretation.

During data cleaning, two extreme outliers were identified and excluded from the sequential multiple data analysis models: Japan, with a meat supply of 54.91 kg/capita/year and a dementia incidence rate of 421.62/100,000 people, and Tonga, with a meat supply of 160.50 kg/capita/year and a dementia incidence rate of 64.31/100,000 people. These outliers were removed from the dataset for analysis.

All variables were compiled and organized using Microsoft Excel® 2016 for subsequent analysis. Each country or population was treated as a unique data point within this ecological analysis framework. The total number of countries analyzed varied for different variables, as comprehensive data were not uniformly available across all indicators due to limitations in reporting by relevant UN agencies.

### 2.2. Statistical analyses

The analysis of the relationship between meat supply and dementia incidence followed a structured, multi-step approach, informed by prior research [28],[29],[31],[37]–[39]:

1. **Initial data exploration**: Scatter plots were generated using Microsoft Excel® 2016 to visually assess the association between global meat supply and dementia incidence. This preliminary step helped identify any extreme outliers and ensured dataset integrity.
2. **Bivariate correlation analysis**: Both Pearson's and nonparametric methods, were conducted to determine the strength and direction of associations among variables including meat supply, dementia incidence, economic status, genetic predisposition, aging, and urbanization.
3. **Partial correlation analysis**: Pearson's partial correlation was used to explore the relationship between meat supply and dementia incidence while statistically controlling for economic status, genetic predisposition, aging, and urbanization as confounding factors.
4. **Multiple linear regression**: A standard (enter) multiple linear regression was performed to delineate the predictive relationship between dementia incidence (dependent variable) and both the main predictor (meat supply) and confounders. This analysis was conducted to evaluate the independent contribution of meat supply in the presence of economic status, genetic predisposition, aging, and urbanization. The regression model quantified the explanatory power of meat supply by comparing results with and without its inclusion as a predictor. Subsequently, stepwise multiple linear regression was applied to identify the most significant predictors of dementia incidence under similar conditions.
5. **Regional correlation analysis**: Bivariate correlations (Pearson's r and nonparametric) were extended to regional groupings to capture variations in the relationship between meat supply and dementia incidence. The analysis stratified countries according to:
  - **World Bank income groups**: High, upper-middle, lower-middle, and low-income countries. Special attention was given to compare high-income countries with combined low- and middle-income countries, addressing the WHO's assertion that over 60% of dementia cases occur in LMICs [40]. Fisher's r-to-z transformation was applied for these comparisons.
  - **UN classification**: Developed versus developing countries, with correlation differences analyzed using Fisher's r-to-z transformation to respond to WHO's regional focus [41].
  - **WHO regional classifications**: Analyses were stratified by regions (Africa, Americas, Eastern Mediterranean, Europe, South-East Asia, and Western Pacific) [42].
  - **Cultural and economic groupings**: Specific country groupings were analyzed, including members of the Asia Cooperation Dialogue (ACD) [43], Asia-Pacific Economic Cooperation (APEC) [44], the Arab World [45], English-speaking countries (based on government data), Latin America [46], Latin America and the Caribbean (LAC) [46], Organization for Economic Co-operation and Development (OECD) [47], and Southern African Development Community (SADC) [48].

Data analysis was conducted using SPSS version 29 (IBM Corp., Armonk, NY, USA) and Microsoft Excel 2016®. The significance level was set at 0.05, with results also reported at 0.01 and 0.001 significance levels. Criteria for stepwise multiple linear regression included a probability of F to enter ≤0.05 and to remove ≥0.10.

### 2.3. Data availability

Details of the data sources are outlined in Section 2. All datasets utilized in this study were freely accessed and downloaded from publicly available resources on UN agency websites. Since the data were sourced from open-access repositories, formal participant consent was not applicable. The use of these datasets aligns with the public use policies specified by the UN agencies, eliminating the need for additional permissions for academic research, as discussed in Section 2 with appropriate references.

### 2.4. Ethics approval

The data utilized in this study were limited to population-level statistics and could not be linked back to any individual, their family, or their community. As such, there was no risk of personal identification or re-identification. The University of Adelaide's Office of Research Ethics, Compliance, and Integrity (ORECI) reviewed and exempted this study from requiring formal ethical approval (ethics approval number: 36289).

## 3. Results

**Figure 1.**
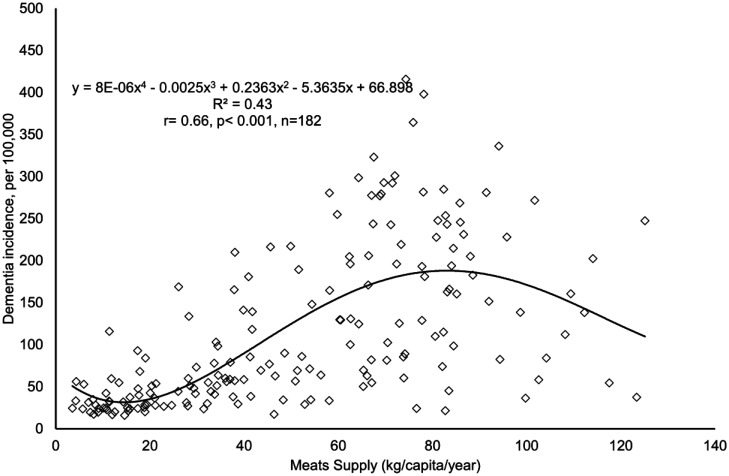
Polynomial correlation plot of total meat supply and dementia incidence. (Data sources and variable definitions: Meat supply, defined as the average annual meat supply per capita (kg/year) over the period 2019–2021, was obtained from the Food and Agriculture Organization. The dementia incidence rate, representing new cases per 100,000 individuals in 2021, was sourced from the Institute for Health Metrics and Evaluation.)

The polynomial correlation plot (Figure 1) reveals a general positive association between total meat supply and dementia incidence across 182 countries. To capture the apparent nonlinear trend more accurately, a fourth-order polynomial regression was applied. The resulting equation (y = 8E–06x^4^ – 0.0025x³ + 0.2363x² – 5.3635x + 66.898) yielded an R² of 0.43, indicating a moderately strong fit. The correlation was statistically significant (r = 0.66, p < 0.001, n = 182), suggesting a robust association between meat supply and dementia incidence on a global scale. Interestingly, the regression curve peaks at approximately 80 kg/capita/year, beyond which dementia incidence appears to decline. This observed inflection point suggests a potential threshold effect.

Table 1 highlights that both Pearson and nonparametric analyses identified significant, strong associations between total meat supply and dementia incidence (r = 0.59 for Pearson and r = 0.68 for nonparametric, p < 0.001). Moreover, moderate to strong significant associations were observed between dementia incidence and factors such as economic affluence, genetic predisposition, aging, and urbanization. These results provided a rationale for considering these variables as potential confounders in the analysis of the link between meat supply and dementia incidence.

**Table 1.** Pearson's r and nonparametric correlation matrix between all variables.

	Meat supply	Dementia incidence	Economic affluence	Genetic predisposition	Ageing	Urban living
Meat supply	1	0.59***	0.62***	0.64***	0.64***	0.56***
Dementia Incidence	0.68***	1	0.60***	0.61***	0.74***	0.50***
Economic Affluence	0.76***	0.78***	1	0.57***	0.733***	0.65***
Genetic Predisposition	0.75***	0.85***	0.89***	1	0.88***	0.52***
Ageing	0.68***	0.829***	0.88***	0.93***	1	0.60**
Urban living	0.58***	0.53***	0.72***	0.63***	0.64***	1

Note: Correlations were evaluated using Pearson's r (above the diagonal) and nonparametric methods (below the diagonal). Statistical significance is denoted as ***p < 0.01, with the number of countries ranging between 176 and 204. Data sources and variable definitions: Meat supply, defined as the average annual meat supply per capita (kg/year) over the period 2019–2021, was obtained from the Food and Agriculture Organization. The dementia incidence rate, representing new cases per 100,000 individuals in 2021, was sourced from the Institute for Health Metrics and Evaluation. The measure for genetic predisposition to dementia was based on the Biological State Index by You & Henneberg (2022) [28]. Economic affluence was represented by per capita GDP, adjusted for purchasing power parity (PPP), as reported by the World Bank (2018) [26]. Aging was assessed through life expectancy at birth, and urbanization was quantified by the proportion of the population residing in urban areas, both sourced from the World Bank (2018) [26].

Partial correlation analysis revealed that total meat supply remained a significant predictor of dementia incidence when economic affluence, genetic predisposition, aging, and urban living were statistically controlled (r = 0.23, p < 0.010, Table 2). Furthermore, when meat supply was treated as a confounding factor, partial correlations indicated that each of the confounding variables (economic affluence, genetic predisposition, ageing, and urban living) remained significantly associated with dementia incidence (r = 0.38, 0.37, 0.59, and 0.26, respectively, p < 0.001). These findings suggest that dementia onset is influenced by multiple interconnected factors.

Standard multiple linear regression (enter) analysis was conducted to predict dementia incidence using meat supply, economic affluence, genetic predisposition, aging, and urban living as predictor variables. When total meat supply was excluded, aging emerged as the only significant predictor of dementia incidence (Beta = 0.79, p < 0.001, Table 3–1). However, when meat supply was included, it became a significant predictor (Beta = 0.20, p < 0.001), along with economic affluence (Beta = 0.08, p < 0.01) and urban living (Beta = −0.02, p < 0.001), although the contributions of economic affluence and urban living were small but statistically significant (Table 3–1).

**Table 2.** Partial correlations of dementia incidence with variables, considering meat supply as both independent variable and confounder.

Variables	Partial correlation to dementia incidence			Partial correlation to dementia incidence		
	r	p	df	r	p	df
Meat supply	0.23	<0.01	170	-	-	-
Economic affluence	-	-	-	0.38	<0.001	173
Genetic predisposition	-	-	-	0.37	<0.001	176
Ageing				0.59	<0.001	178
Urban living	-	-	-	0.26	<0.001	178

Note: Data sources and variable definitions: Meat supply, defined as the average annual meat supply per capita (kg/year) over the period 2019–2021, was obtained from the Food and Agriculture Organization. The dementia incidence rate, representing new cases per 100,000 individuals in 2021, was sourced from the Institute for Health Metrics and Evaluation. The measure for genetic predisposition to dementia was based on the Biological State Index by You & Henneberg (2022) [28]. Economic affluence was represented by per capita GDP, adjusted for purchasing power parity (PPP), as reported by the World Bank (2018) [26]. Aging was assessed through life expectancy at birth, and urbanization was quantified by the proportion of the population residing in urban areas, both sourced from the World Bank (2018) [26]. - Included as the confounding factor.

In the stepwise multiple linear regression analysis, aging was again identified as the only significant predictor of dementia incidence when meat supply was excluded, yielding an adjusted R² of 0.54 (Table 3–2). Once meat supply was included, it became the second most significant predictor, increasing the adjusted R² to 0.56. Genetic predisposition was identified as the third most important factor, while economic affluence and urban living remained non-significant in the stepwise model.

A standard multiple linear regression (enter method) was conducted to predict dementia incidence using meat supply, economic affluence, genetic predisposition, aging, and urban living as predictors. When meat supply was excluded, aging was the only significant predictor of dementia incidence (β = 0.79, p < 0.001, Table 3–1). However, when meat supply was included, it became a significant predictor (Beta = 0.202, p < 0.001), along with economic affluence (beta = 0.08, p < 0.010) and urban living (Beta = −0.02, p < 0.001), though the contributions of economic affluence and urban living were small but statistically significant (Table 3–1).

In the stepwise multiple linear regression analysis, aging remained the only significant predictor of dementia incidence when meat supply was excluded, yielding an adjusted R^2^ of 0.54 (Table 3–2). When meat supply was added, it emerged as the second most significant predictor, raising the adjusted R^2^ to 0.56. Genetic predisposition was identified as the third predictor, while economic affluence and urban living remained non-significant in the stepwise model.

**Table 3.** Results of multiple linear regression analyses to sort significant predictors of dementia incidence.

Table 3–1: ENTER Variables entered		Excluding meat supply		Including meat supply	
		Beta	Sig.	Beta	Sig.
Meats intake		Not added	Not applicable	0.20	<0.001
Economic affluence		0.06	0.45	0.08	<0.01
Genetic predisposition		−0.14	0.20	−0.18	0.34
Ageing		0.79	<0.001	0.72	0.11
Urban living		0.04	0.53	−0.02	<0.001

Table 3–2: STEPWISE		Meat supply not added		Meat supply added	

Rank	Variables entered	Adjusted R square	Rank	Variables entered	Adjusted R square

1	Ageing	0.54	1	Ageing	0.54
	Meats intake	Not significant	2	Meats intake	0.56
	Genetic predisposition	Not significant	3	Genetic predisposition	0.57
	Economic affluence	Not significant		Economic affluence	Not significant
	Urban living	Not significant		Urban living	Not significant

Note: Stepwise multiple linear regression modelling is reported. Contribution of variables is listed in order of how much they contribute to dementia incidence. Data sources and variable definitions: Meat supply, defined as the average annual meat supply per capita (kg/year) over the period 2019–2021, was obtained from the Food and Agriculture Organization. The dementia incidence rate, representing new cases per 100,000 individuals in 2021, was sourced from the Institute for Health Metrics and Evaluation. The measure for genetic predisposition to dementia was based on the Biological State Index by You & Henneberg (2022) [28]. Economic affluence was represented by per capita GDP, adjusted for purchasing power parity (PPP), as reported by the World Bank (2018) [26]. Aging was assessed through life expectancy at birth, and urbanization was quantified by the proportion of the population residing in urban areas, both sourced from the World Bank (2018) [26].

Table 4 displays the bivariate correlations examining the relationship between meat supply and dementia incidence across different country classifications. Overall, the analysis found positive correlations between meat consumption and dementia rates across most country categories, evaluated using both Pearson and nonparametric tests. The observed correlation strength and statistical significance varied, potentially influenced by differences in sample sizes and the level of homogeneity within each group. Fisher's r-to-z transformation analysis indicated that the association between meat supply and dementia incidence was notably stronger in low- and middle-income countries than in high-income nations (z = 3.9, p < 0.001 for Pearson's r; z = 5.6, p < 0.001 for the nonparametric analysis). Additionally, the correlation was significantly greater in developing countries compared to developed ones (z = 2.5, p < 0.010 for Pearson's r; z = 3.9, p < 0.001 for the nonparametric analysis). These results point to a potentially more substantial influence of meat supply on dementia incidence in lower-income regions compared to higher-income regions (Table 4).

**Table 4.** Meat supply correlated to dementia incidence in various country groupings.

Country groupings	Pearson r	p	Nonparametric	p	n
Worldwide	0.59	<0.001	0.68	<0.001	182
UN common practice					
Developed countries	0.10	0.53	−0.01	0.98	44
Developing countries	0.50	<0.001	0.60	<0.001	138
Fisher r-to-z transformation	Developing vs. Developed: z = 2. 5, p < 0.010		Developing vs. Developed: z = 3.94, p < 0.001		
World Bank income classifications					
High-income (HI) countries	−0.07	0.60	−0.130	0.35	54
Low-income (LI) countries	0.18	0.35	0.240	0.23	28
Low-middle-income (LMI) countries	0.37	<0.010	0.48	< 0.001	49
Upper-middle-income (UMI) countries	0.20	0.17	0.26	0.07	51
Low- and middle-income countries (LI, LMI, UMI)	0.52	<0.001	0.67	<0.001	128
Fisher r-to-z transformation	Low- and middle-income vs. high: z = 3.92, p < 0.001		Low- and middle-income vs. high: z = 5.64, p < 0.001		
WHO regions				
African region countries	0.59	<0.001	0.45	<0.010	45
American region countries	0.58	<0.001	0.59	<0.001	35
Eastern Mediterranean region countries	−0.10	0.68	0.11	0.63	21
European region countries	0.47	<0.001	0.35	<0.05	50
South-East Asian region countries	0.20	0.59	0.32	0.37	10
Western Pacific region countries	0.30	0.18	0.18	0.43	21
Countries grouped with various factors					
Asia Cooperation Dialogue (ACD)	0.15	0.46	0.010	0.99	27
Asia-Pacific Economic Cooperation (APEC)	0.66	<0.010	0.70	<0.010	17
Arab World	−0.19	0.42	−0.06	0.79	21
English as official language (EOL)	0.65	<0.001	0.78	<0.001	51
Latin America (LA)	0.64	<0.001	0.72	<0.001	23
Latin America and the Caribbean (LAC)	0.49	<0.010	0.54	<0.001	33
Organization for Economic Cooperation and Development (OECD)	−0.04	0.84	−0.14	0.41	36
Southern African Development Community (SADC)	0.69	<0.01	0.55	<0.05	16

Note: Bivariate correlations (Pearson r and nonparametric) between meat supply and dementia incidence within country groupings were reported. Data sources and variable definitions: Meat supply, defined as the average annual meat supply per capita (kg/year) over the period 2019–2021, was obtained from the Food and Agriculture Organization. The dementia incidence rate, representing new cases per 100,000 individuals in 2021, was sourced from the Institute for Health Metrics and Evaluation.

## 4. Discussion

This study explored the global relationship between total meat supply and dementia incidence, broadening the focus beyond red meat to assess the collective impact of both red and white meat on cognitive health. Results indicate that total meat supply is a significant predictor of dementia incidence, even after controlling for key factors such as economic status, genetic predisposition, aging, and urbanization. This suggests that both red and white meats, each contributing unique compounds potentially affecting cognitive health, may collectively influence dementia risk.

The findings of this study demonstrate a positive global correlation between total meat supply and dementia incidence, consistent with previous research linking high meat consumption to adverse health outcomes. In particular, our results align with ecological and observational studies that have examined the association between meat supply and Alzheimer's disease [49],[50], which accounts for the majority of dementia cases. These studies provide cross-national evidence that dietary factors, including meat consumption, may contribute to Alzheimer's risk through mechanisms such as chronic inflammation, oxidative stress, and impaired vascular function.

The study's emphasis on total meat supply contributes valuable insights into dietary patterns and their broader implications for cognitive health. Both red and white meats contain compounds, such as L-carnitine and heme iron that may contribute to inflammatory processes and oxidative stress [51], which are well-documented mechanisms in dementia development. Although white meat is often considered healthier due to lower saturated fat content [52], recent evidence suggests it may also pose cognitive health risks through compounds like trimethylamine-N-oxide (TMAO) [53], which is linked to neuroinflammation and may play a role in dementia progression [54].

The current literature on meat consumption and dementia risk remains varied, with mixed findings from cohort studies. For example, a UK Biobank cohort study found that processed meat was associated with an increased risk of dementia, while unprocessed red meat was linked to a reduced risk [55]. In contrast, the Three-City (3C) cohort study, conducted in France, reported that lower meat consumption (≤1 time per week) was linked to higher dementia risk compared to higher consumption (≥4 times per week), though differences in methodology and categorization could explain these inconsistencies [56]. Similarly, a French cohort study observed a non-significant association between infrequent meat consumption and dementia incidence, likely due to small sample sizes [57]. In a German cohort, no significant relationship was found between meat consumption and dementia risk over four years, though this study examined specific meat items only [58]. Such inconsistencies point to the need for further research clarifying the effects of different meat types and consumption levels on dementia risk.

Variations in meat composition may partially explain these divergent findings. Compounds commonly found in processed meats, such as nitrites and N-nitroso compounds, are known to promote oxidative stress, lipid peroxidation, and inflammation, all of which may contribute to dementia [59]. Additionally, increased meat consumption is associated with higher intake of saturated fatty acids, which have been linked to dementia risk [60]. Processed meats also contain high sodium levels, which could adversely impact cognitive health. Our findings align with emerging global evidence from 2025 suggesting that red meat supply is positively associated with dementia risk [12], while diets rich in plant-based foods and fish, such as the Mediterranean and Nordic diets, appear to be protective [13],[61]. These dietary models emphasize the role of unsaturated fats and reduced saturated fat intake. Both of these factors may mediate inflammation and neurodegenerative processes. Studies in animal models show that high-salt diets can elevate blood pressure and reduce cerebral blood flow, potentially impairing cognitive function [62].

Beyond inflammation and metabolic syndrome, cholesterol regulation represents another plausible pathway linking meat consumption to cognitive decline. Animal products are known to elevate low-density lipoprotein cholesterol (LDL-C), while plant-based foods generally reduce it. Elevated LDL-C has been associated with increased risk for both cardiovascular disease and Alzheimer's disease. Studies have shown that phytochemicals and plant-based whole foods can lower LDL-C through beneficial molecular mechanisms [63], and that aggressive LDL-C lowering may influence dementia risk [64]. A meta-analysis also reported elevated total cholesterol and LDL-C levels in individuals with Alzheimer's disease and mild cognitive impairment [64]. Furthermore, recent systematic reviews highlight associations between meat product consumption and increased risks for both Alzheimer's dementia and type 2 diabetes, two conditions often linked through vascular and metabolic pathways [64]. These findings support the possibility that cholesterol modulation may partially mediate the observed meat–dementia association in this study.

Distinctions between processed and unprocessed meats may help clarify why processed meats are more consistently associated with dementia risk compared to unprocessed meats, such as poultry and red meat. Conversely, high protein intake from meat could potentially protect against dementia, as adequate protein levels have been associated with reduced cognitive impairment risk [65]. Age-related iron accumulation in the brain, however, may increase neurodegeneration risk, with abnormal iron metabolism inducing oxidative stress, a contributing factor in cognitive decline [66]. Some studies even suggest that iron in red meat supports brain health by reducing iron deficiency, a condition associated with cognitive decline. This further complicated the relationship between meat consumption and dementia [67].

High meat consumption has been associated with an increased risk of obesity, cardiovascular disease, and type 2 diabetes mellitus (T2DM) [68], which are known metabolic conditions that may mediate the relationship between diet and cognitive decline [69],[70]. Although the causal link between T2DM and dementia remains debated, these conditions contribute to vascular dysfunction, neuroinflammation, and insulin resistance—mechanisms implicated in dementia pathogenesis [71]. Therefore, the observed association between meat supply and dementia incidence may be partially explained by these intermediary metabolic disorders. Our regression model accounted for 56.3% of the variance in dementia incidence, suggesting that additional unmeasured factors may influence dementia risk. In ecological and population-level research, adjusted R² values above 0.5 are generally considered acceptable [72]–[74]. Nonetheless, this limitation underscores the need for future studies to incorporate other potential predictors, such as genetic predisposition, early-life exposures, and lifestyle variables.

The association between meat supply and dementia incidence was more pronounced in low- and middle-income countries than in high-income countries. This discrepancy may reflect differences in dietary diversity, plant-based food consumption, and healthcare access, which can mitigate or amplify the adverse effects of meat consumption on cognitive health. In regions with limited dietary variety and health resources, high-meat diets may compound dementia risk. Notably, prior research has shown that the correlation between meat supply and Alzheimer's disease becomes stronger with longer follow-up periods [10], highlighting the importance of accounting for latency in dietary exposures. This supports our use of multi-year averages (2019–2021) to better reflect medium-term dietary patterns relevant to dementia development.

Our regression analysis identified aging as the strongest predictor of dementia incidence, affirming the well-established role of aging as the primary non-modifiable risk factor for dementia. However, total meat supply also emerged as a secondary predictor, underscoring its potential as a modifiable factor that could be addressed in dementia prevention strategies. Although factors like economic status and urbanization were also significant, their contributions to dementia risk were relatively minor, suggesting that broader lifestyle factors, including dietary choices, healthcare access, and physical activity may indirectly influence dementia risk. These findings support the need for comprehensive, multifaceted approaches to dementia prevention that address modifiable risk factors, including dietary intake.

The fourth-order polynomial regression revealed a nonlinear association between total meat supply and dementia incidence, with rates peaking at approximately 80 kg/capita/year before showing a modest decline. Although this pattern may suggest a potential threshold effect, it should be interpreted cautiously due to the limited number of observations at higher supply levels and the possibility of unmeasured confounders. Additional research is necessary to determine whether this turning point reflects a genuine biological mechanism. However, given the extensive subgroup analyses already conducted across regional and economic classifications, further regression analyses within narrower supply ranges are unlikely to meaningfully change the overall conclusions.

## 5. Limitation of this study

First, the cross-sectional ecological design of this study precludes the ability to establish causality or temporal sequence. The use of recent meat supply data (2019–2021) alongside dementia incidence figures from 2021 does not account for the decades-long latency period typically associated with dementia development. Dietary exposures occurring 20 to 30 years earlier would be more relevant for assessing long-term risk. Furthermore, reliance on population-level data may obscure individual-level variability in dietary habits, lifestyle factors, and health outcomes, introducing the potential for ecological fallacy. Associations observed at the national level may not necessarily reflect relationships at the individual level.

Second, dementia has a long latency period, and its development may be influenced by dietary patterns sustained over many years or even decades. The use of meat supply data from 2019 to 2021 represents medium-term exposure and may not fully capture long-term dietary influences on dementia risk. This limitation is inherent in ecological analyses where historical dietary datasets with global coverage are often unavailable.

Third, dementia incidence data were sourced from international databases, such as IHME, which may be incomplete, particularly in developing nations with limited record-keeping. Although controls for economic affluence, genetic predisposition, aging, and urbanization were applied, some residual bias might persist.

Fourth, meat supply was broadly defined as “flesh of animals”, without accounting for the effects of processing and cooking methods, which can influence health outcomes. Additionally, the study used FAO data on general meat supply, not direct human consumption, making meat supply an approximation rather than a precise measure.

Fifth, other influential factors, such as physical activity and healthcare access, were not directly addressed. Future research should use longitudinal data to assess causality and examine whether reducing meat supply could lower dementia risk across diverse contexts. These limitations highlight the need for caution in interpreting results and the importance of further research to confirm these findings.

Finally, due to the ecological nature of the data, this study could not determine dose-response relationships or establish safe thresholds for meat consumption. Meat subtypes (e.g., red vs. white, processed vs. unprocessed) were not distinguished, and potential interactions with other dietary components were not examined. These limitations constrain the ability to draw specific dietary recommendations and underscore the need for longitudinal, individual-level studies. Additionally, the use of FAO food supply data likely overestimates actual intake, as it reflects availability rather than consumption, excluding losses from waste and spoilage.

## 6. Conclusions

Globally, access to total meat (flesh) could play a critical role in predicting dementia risk, particularly within low- and lower-middle-income nations. This study contributes to understanding dietary risk factors for dementia by examining total meat supply rather than focusing solely on red meat. The study findings suggest that high total meat consumption may increase dementia risk, particularly in low- and middle-income countries, highlighting the importance of considering total dietary intake in public health strategies and dietary recommendations for cognitive health.

## Use of AI tools declaration

The author declares that an Artificial Intelligence (AI) tool, ChatGPT (OpenAI, GPT-4), was used in the preparation of this article for the sole purpose of language refinement. Specifically, the AI tool assisted in improving grammar, enhancing sentence clarity, and ensuring consistency in tone and academic writing style. It was not used to generate original content, interpret findings, conduct data analysis, or contribute to any scientific reasoning or conclusions. Language editing support was applied to the Abstract, Introduction, Discussion, and Limitations sections to enhance readability and coherence. All substantive content, including the study design, data analysis, results interpretation, and conceptual development, was entirely produced by the author.
